# Seasonal contact and migration structure mass epidemics and inform outbreak preparedness in a vulnerable marine mammal

**DOI:** 10.1098/rspb.2025.0698

**Published:** 2025-07-30

**Authors:** Melissa Ann Collier, Kim Urian, Sarah Theisen, Ann-Marie Jacoby, Sarah Wilkin, Eric M. Patterson, Megan Wallen, Vittoria Colizza, Janet Mann, Shweta Bansal

**Affiliations:** ^1^ Department of Biology, Georgetown University, Washington, DC, USA; ^2^ Duke University Marine Laboratory, Beaufort, NC, USA; ^3^ NOAA Fisheries, Silver Spring, MD, USA; ^4^ NOAA Fisheries West Coast Region, Seattle, WA, USA; ^5^ Institut Pierre Louis d’Epidémiologie et de Santé Publique, INSERM, Sorbonne Université, Paris F75012, France; ^6^ Department of Psychology, Georgetown University, Washington, DC, USA

**Keywords:** disease model, metapopulation, marine mammal, dolphin, migration

## Abstract

Infectious diseases have detrimental impacts across wildlife taxa. Despite this, we often lack information on the complex spatial and contact structures of host populations, reducing our ability to understand disease spread and our preparedness for epidemic response. This is also prevalent in the marine environment, where rapid habitat changes due to anthropogenic disturbances and human-induced climate change are heightening the vulnerability of marine species to disease. Recognizing these risks, we leveraged a collated dataset to establish a data-driven epidemiological metapopulation model for Tamanend’s bottlenose dolphins (*Tursiops erebennus*), whose populations are periodically impacted by deadly respiratory disease. We found their spatial distribution and contact is heterogeneous throughout their habitat and by ecotype, which explains differences in past infection burdens. With our metapopulation approach, we demonstrate spatial hotspots for epidemic risk during migratory seasons and that populations in some central estuaries would be the most effective sentinels for disease surveillance. These mathematical models provide a generalizable, non-invasive tool that takes advantage of routinely collected wildlife data to mechanistically understand disease transmission and inform disease surveillance tactics. Our findings highlight the heterogeneities that play a crucial role in shaping the impacts of infectious diseases, and how a data-driven understanding of these mechanisms enhances epidemic preparedness.

## 1. Introduction

Infectious diseases have detrimental impacts on wildlife [1,2], but they remain understudied in marine populations despite their significant impact on these ocean-dwelling species. The effect of disease on cetaceans (whales, porpoises and dolphins) is of particular interest, as they are predators and can serve as sentinel species; their protection is vital for maintaining a balanced and healthy ocean ecosystem [3–5], and their populations are currently under threat of intensified pathogen outbreaks due to the direct and indirect effects of climate change [6]. However, few data exist on the spatial and social structures of these species [7,8], consequently limiting our ability to analyse, forecast and respond to disease threats. To address this, we leverage multiple individual-level datasets on the behaviour of a sentinel cetacean species that is periodically impacted by deadly respiratory disease. We develop a mathematical modelling approach to (i) characterize how sociality and space use impact the structure of vulnerability in a complex marine population and (ii) inform preparedness for future deadly outbreaks in these vulnerable and understudied taxa.

Respiratory pathogens have contributed to wildlife population declines in species including bighorn sheep [9], mountain goats [10], house finches [11], lizards [12] and tortoises [13], due to their rapid spread via direct and indirect transmission routes [14]. The respiratory transmitted morbilliviruses are especially deadly in marine environments [15,16] with dolphin morbillivirus (DMV) being the most devastating among delphinid populations [16]. For instance, DMV caused more than 1000 striped dolphins (*Stenella coeruleoalba*) deaths in Spain, Italy and France in the 1990s [17], with resurging outbreaks in 2007 and 2011 [18,19]. The most severe DMV outbreaks on record occurred in Tamanend’s bottlenose dolphins (*Tursiops erebennus*, formerly *Tursiops truncatus* [20]) along the United States Atlantic coast where populations declined by over 50% in 1987 [21] and 2013 [22], both declared ‘unusual mortality events’. To forecast outbreaks and develop surveillance for vulnerable species like Tamanend’s dolphins, it is crucial to understand the spatial distribution and contact structure driving respiratory pathogen transmission in wildlife.

Animal movements such as dispersal (short-distance habitat shifts) and migration (long-distance repeated seasonal movements) facilitate pathogen spread within and across species [7]. Increased collection of marine mammal stranding data has improved our understanding of respiratory disease distribution in marine environments [15,16,23]. Because pathogens rely on host movement to disperse, spatiotemporal behaviour will also shape host exposure and transmission risk [24]. However, without such data, predicting pathogen distribution and spread remains a challenge. For example, Tamanend’s dolphins comprise multiple populations along the US Atlantic Coast from New York and Florida that overlap in space and time [25], though the extent of this overlap remains unclear. Studies in Georgia [26] and Florida [27] show seasonal shifts and spatial overlap among populations, but these are limited to near-shore regions and do not represent their full habitat range. These constraints reduce our ability to understand or predict disease outbreaks.

While host space use determines a pathogen’s distribution, its transmission within populations is driven by host contact. Thus, social systems [8] and individual heterogeneities in contact behaviour [28,29] shape disease dynamics. Cetacean social structures vary widely from relatively solitary (e.g. baleen whales) to highly gregarious (e.g. dolphins) [30] suggesting subsequent variability in disease transmission. Tamanend’s dolphins are highly social [31] and engage in synchronized breathing (simultaneous close-proximity surfacing [32]) to build lifelong social bonds [33–35], while also increasing their risk of respiratory pathogen transmission [8,16,36]. Until recently, little was known about the structure of this contact behaviour in Tamanend’s dolphins, but new findings show it varies by age and sex, with implications for infection risk [36]. However, our ability to predict seasonal variation in disease risk due to social behaviour remains limited.

**Figure 1.**
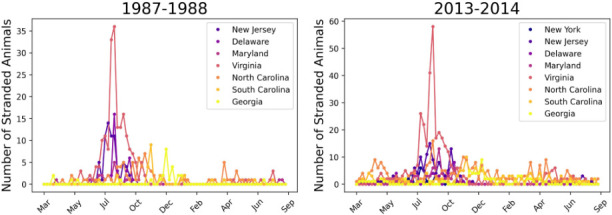
Bottlenose dolphin strandings detected during two DMV outbreaks along the US Atlantic coastline. During both the epidemics beginning in 1987 (left) and 2013 (right), confirmed strandings (when a sick, injured, or dead dolphin is found floating or washed ashore) of dolphins peaked in July and August along the northern part of the US Atlantic coastline, with smaller peaks in the south in October–December. While there is notable spatial heterogeneity in strandings along the coastline during both outbreaks, the spatiotemporal distribution of infections was remarkably similar between the two epidemics.

Despite past epidemics motivating interest in Tamanend’s dolphins' spatial and contact dynamics, the complexity of these processes has hindered understanding of their role in disease spread. The National Oceanic and Atmospheric Administration (NOAA) manages Tamanend’s dolphins as ‘stocks’ along the US eastern seaboard from New York to Florida [25] comprising two main ecotypes: ‘estuarine’ stocks that inhabit nearshore bays and rivers with small range dispersal, and ‘coastal’ stocks that occupy deeper ocean waters, and show broader migratory patterns [25]. DMV burden has historically varied across latitude and ecotype. In the past two outbreaks, most infections occurred at northern latitudes in warmer months, while southern regions experienced later, lower, infection peaks [37,38] (figure 1). Coastal stocks were also heavily impacted [21,39], while estuarine stocks were seemingly less impacted [40]. Only one study has examined DMV spatial spread in this species, which estimated key parameters like the basic reproduction number (R0) and infectious period. However, a significant gap persists as no mechanistic framework exists to capture spatial heterogeneity and inform ecotype-specific surveillance and preparedness in systems where standard interventions are logistically or legally unfeasible.

Given their complex socio-spatial dynamics, epidemiological models driven by movement and contact behaviours collected along the entire habitat range of bottlenose dolphins are needed. Metapopulation models provide an approach to model disease dynamics in spatially separated but connected populations to answer diverse questions related to pathogen invasion dynamics, persistence, control measures, and evolution [41]. Successful models applied in wildlife systems have described the persistence of lyssavirus [42] and spread of white-nose syndrome [43] in bat species, the spread of bovine tuberculosis among the migratory brushtail possum [44], and the prevalence of sarcoptic mange and canine distemper in Yellowstone wolves [45]. To our knowledge, epidemiological metapopulation inference has only been applied in one marine mammal system, to determine persistence thresholds for respiratory viruses in harbour seals [46], with none applied in cetaceans.

We present the first data-driven metapopulation model for cetacean respiratory disease, applied to Tamanend’s bottlenose dolphins along the US Atlantic coast. To parameterize this model and overcome past limitations, we (i) leveraged a collated dataset of Tamanend’s dolphin sightings from 28 field sites (New Jersey to Georgia) and (ii) collected synchronized breathing contact data for both coastal and estuarine ecotypes at one site. We validated the model using 1987 and 2013 DMV outbreak data, then explored the roles of seasonality, environment and social behaviour on disease dynamics. Finally, we applied our best model along the Tamanend’s dolphin habitat range, to assess epidemic risk and identify potential sentinel surveillance sites. Our work highlights how metapopulation structure influences disease risk and illustrates how such models can enhance preparedness for future outbreaks in vulnerable wildlife.

## 2. Methods

We developed an epidemiological model to assess how Tamanend’s bottlenose dolphin metapopulation structure influenced past DMV outbreaks (figure 1). Their US Atlantic habitat (New Jersey to Georgia) was divided into ecologically relevant metapopulation ‘patches’. The Florida coastline was excluded due to inconsistent sighting data but analysed theoretically (electronic supplementary material, §S10).

We accounted for differences between the two main ecotypes—coastal (long-distance migration) and estuarine (short-distance dispersal) [25]—using the broader term 'movement' for both. Because NOAA stock boundaries do not fully reflect recently observed seasonal and spatial heterogeneity, we defined patches using empirical data rather than relying solely on stock designations, but details on NOAA’s estimates of ecotype structure, movement and size can be found in the electronic supplementary material, §S1.

**Figure 2.**
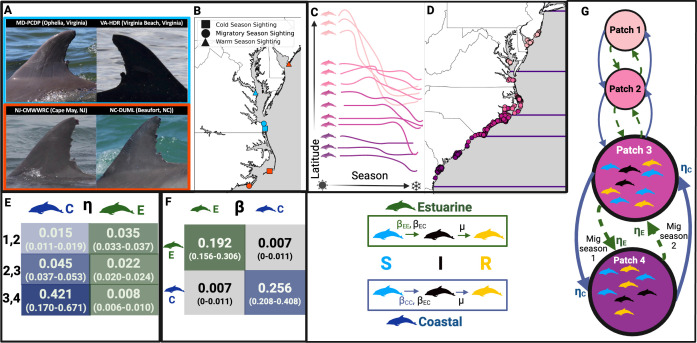
Methods and structure of the epidemiological metapopulation model for the Tamanend’s bottlenose dolphins. (A) We matched photos of dorsal fins across 28 photo-ID catalogues from New Jersey to Georgia to (B) establish sighting histories for 423 individuals in the warm water, migratory and cold-water seasons. (C) These sighting histories are transformed into individual time series of sighting locations over the course of a year (represented here visually as lines indicating latitudinal location by season by individual) which we algorithmically group into four clusters. (D) Using these cluster assignments and all the warm-water season sightings of individuals from the catalogues (points, coloured by cluster), we establish four ecologically relevant metapopulation patches (purple lines). (E) The average rates (and 95% CI) of migration (coastal ecotype, blue) or dispersal (estuarine ecotype, green) between the patches (known as $\eta_{s,pq})$ are determined by fitting a multistate capture–recapture model to individual sighting histories. (F) The average transmission rates ($\beta_{ss^{\prime }}$) and their 95% CI for within and between ecotypes are established with synchronized breathing contact field data. (G) The resulting epidemiological metapopulation SIR model allows individuals to move between patches based on $\eta_{s,pq}$, while within each patch they can move from susceptible (S) to infected (I) based on $\beta_{ss^{\prime }}$, and recover at recovery rate μ. Created in BioRender (https://BioRender.com/ny8vxau).

We estimated movement rates between patches and contact-driven DMV transmission rates within patches, incorporating both into our metapopulation model (figure 2). To validate our model, we compared infection predictions to 1987 and 2013 DMV outbreak data and estimated infection burden by ecotype to assess alignment with expert views that coastal ecotypes were more affected [22,25,47]. Finally, we used our model to evaluate preparedness to future DMV outbreaks.

### (a) Metapopulation structure inference

#### (i) Defining patches and movement rates

#### Dataset

The Mid-Atlantic Bottlenose Dolphin Catalog (MABDC) [48] is a cooperative photo-ID database maintained by a curator containing dorsal fin images of bottlenose dolphins collected along the US Atlantic coast by various researchers. Since dolphins have uniquely marked dorsal fins, these images can be used in mark-recapture tracking of individuals across time and space [49]. Hosted on OBIS-SEAMAP (https://seamap.env.duke.edu), the MABDC also allows researchers to match dorsal fins across catalogues and infer seasonal movement.

We generated sighting histories by comparing dorsal fin photographs (figure 2A) across 28 MABDC fin catalogues from New Jersey to Georgia. We completed 215 of 377 possible pairwise comparisons (see electronic supplementary material, §S2 for details). We compiled annual sighting histories for all dolphins in these comparisons (figure 2B) assuming movement patterns are stable across years and focusing only on seasonal presence. Inclusion criteria required individuals to have (i) at least five sightings and (ii) at least one sighting in the warm-water season (July–September), cold-water season (January–March) and one migratory season (April–June or October–December) (*n* = 423). Seasons were based on 20 years of sea surface temperature data (electronic supplementary material, §S3, figure S2).

#### Defining metapopulation patches

First, we divided the coastline into ecologically relevant metapopulation patches that dolphins move between during an annual period. Since NOAA considers unique dolphin stocks to be composed of individuals with similar seasonal ranges, we performed a hierarchical clustering analysis (Ward’s method) on the spatiotemporal time series of our 423 dolphins to predict natural groupings according to sighting histories (figure 2C). We identified the optimal number of clusters, P, to be when the size of the clusters are most uniform (satisfying biological, methodological and modelling considerations) while their silhouette scores (mean distance between clusters) are greater than the average score of the full dataset (electronic supplementary material, figure S3, §S4.1).

Sightings of individuals in each cluster in the warm-water season, when stocks are thought to overlap the least [25] were plotted, with the resulting ranges used to divide the coastline into P patches (figure 2D). These patches were assumed to be the optimal areas from which individuals of each cluster would leave once temperatures begin to decrease and return to when temperatures begin to increase. We validated the patch configuration by comparing them to the NOAA estimated ranges for bottlenose dolphin stocks in this region (electronic supplementary material, figure S1).

#### Determining movement rates between patches by ecotype

The comparison of our patch configuration to NOAA’s stock ranges (electronic supplementary material, figure S1) suggested that estuarine ecotypes occupy one patch year-round or disperse between a maximum of two patches, whereas migratory coastal ecotypes migrate between three or more patches over the course of a year. Therefore, we classified individuals sighted in one or two patches in the MABDC as estuarine (*n* = 394) and those sighted in three or more patches as coastal (*n* = 29).

Next, we estimated movement rates across patches for both estuarine and coastal ecotypes. Due to considerable variability in the sampling effort across the field sites that contribute to the MABDC, traditional capture–recapture models are not realistic. To analyse these data, we fit a continuous time multistate capture–recapture model that accounts for stochasticity in detection times [50]. Specifically, we fit models to both coastal and estuarine individuals and estimated the transition rate ($m_{pq,s}$) between patch p and q, for each ecotype s. For the estuarine model, we only included estuarine individuals observed in more than one patch (*n* = 220). Additional details of this model are in the electronic supplementary material, §S5.

We then calculated the movement rate between patch p and q for ecotype s ($\eta_{pq,s}$) as:


$$\eta_{\;pq,\;s}\;=\;m_{pq,\;s}n_{p,\;s}n_{q,\;s}$$

where $n_{p,s}$ and $n_{q,s}$ are the proportions of individuals of ecotype s in patch p and q, respectively (electronic supplementary material, table S4) that have been seen moving to another patch as observed in the MABDC (figure 2E).

#### (ii) Defining contact-driven transmission rates by ecotype

#### Data Collection

To estimate the rate of contact-driven transmission in bottlenose dolphins, we used behavioural data collected from boat-based observational surveys and focal animal sampling [51] from one research site that contributes to the MABDC, the Potomac Chesapeake Dolphin Project (PCDP). The PCDP collects data in the Potomac River and middle Chesapeake Bay and individuals seen at this field site have been established by additional sightings in the MABDC to be both estuarine and coastal ecotypes. Given this and the robust similarities among *Tursiops* populations, we assumed that data on contact rates obtained from this field site are representative of social contact for both ecotypes of Tamanend’s dolphins.

Since DMV is a respiratory-transmitted pathogen, we focused on synchronized breathing interactions as our measure of contact and collected the number of synchronized breaths continuously throughout a focal follow for the focal individual [49]. We collected 101 follows on 99 individuals between June and September 2015–2022 with an average time of 25 minutes.

#### Determining coastal and estuarine ecotypes for focal individuals

As none of our 101 focal PCDP individuals were sighted at another location in the MABDC, we used a different approach to classify each individual as estuarine or coastal ecotype [52]. We used a K-means clustering algorithm to cluster focal individuals based on the average distance each individual was sighted from shore which resulted in three clusters: a ‘nearshore’ cluster (*n* = 55 follows), a ‘midshore’ cluster (*n* = 30 follows) and a ‘farshore’ cluster (*n* = 16 follows). Based on expert opinion [25,53], we designated individuals in our nearshore cluster as estuarine and individuals in the farshore cluster as coastal (see electronic supplementary material, §S6.1 for more methodological details). Individuals in the midshore cluster were classified as ‘undetermined’ and therefore dropped from further analysis.

Using our focal follow data, we estimated average daily synchrony degree for both estuarine and coastal ecotypes, which represents the number of unique individuals an animal has synchronous breathing contact with over an average day for ecotype s, $k_{s}$, based on methodology adapted from past work [36] (see electronic supplementary material, §S6.2).

#### *Calculating transmission rate* β

We estimated the transmission rates for DMV within each ecotype s, $\beta_{ss}$ (figure 2F) based on the average synchrony degree by ecotype, $k_{s}$ and the per contact infectiousness of DMV (τ = 0.032; calculated in the electronic supplementary material, §S7.1):


$$\beta_{ss}=k_{s}\tau$$

We also calculated a range for $\beta_{ss^{\prime }}$ (figure 2F), the transmission rate between ecotypes s and $s^{\prime }$ as:


$$\beta_{ss^{\prime }}=\;k\tau \alpha$$

based on the average synchrony degree across both ecotypes, k, and the degree of mixing between ecotypes (α) estimated to vary between 0 and 6% from PCDP sighting data (see electronic supplementary material, §S7.2).

### (b) Epidemiological metapopulation model

Using our inferred metapopulation structure, we modelled the spread of DMV in Tamanend’s dolphins between New Jersey and Georgia. Each patch, p, (where pϵ{1,2,3,4}) was populated with individuals of both ecotypes s, (where sϵ{C,E}) based on the best estimates of NOAA stock assessments from before the 2013 DMV epidemic (see electronic supplementary material, §S4.2). We also performed a sensitivity analysis on the estuarine ecotype patch sizes due to uncertainty in their empirical estimates (see electronic supplementary material, §S11.1). To represent migration, coastal dolphins move among patches (based on $\eta_{pq,C}$) during the first (March–June) and second (October–December) migratory season and did not move in the warm (July–September) or cold (January–February) water season. To represent dispersal, estuarine dolphins move (based on $\eta_{pq,E}$) a maximum of one patch south between January and June and one patch north between July and December.

Within each p, for each ecotype s, we considered a standard SIR model to simulate the spread of DMV through a patch (figure 2G) and considered frequency-dependent transmission for DMV [22]: susceptible dolphins (S) became infectious (*I*) and able to transmit DMV based on a force of infection $\lambda_{p,s}$ and became removed (R) with removal rate *µ* based on the infectious period of DMV (5–10 days) [22]. $\lambda_{p,s}$ is described as:


$$\lambda_{p,s}\;=\;\sum_{s^{\prime }}\beta_{ss^{\prime }}\frac{I_{ps^{\prime }}}{N_{ps^{\prime }}}$$

where I is the number of currently infected dolphins in p of ecotype s, and N is the total number of dolphins in p of ecotype s.

We thus model the progression of disease using a discrete and stochastic implementation of the following model [42] (see electronic supplementary material, §S8). We completed 100 simulations of DMV spread using this model.


$$\frac{dS_{p,s}}{dt}\;=\;-\lambda_{p,s}S_{p,s}+\;\sum_{q}^{}\eta_{pq,\;s}S_{q,s}\;-\;\sum_{q}^{}\eta_{qp,\;s}S_{p,s};\;\frac{dI_{p,s}}{dt}\;=\;\lambda_{p,s}S_{p,s}-\mu I_{p,s}+\;\sum_{q}^{}\eta_{pq,\;s}I_{q,s}\;-\;\sum_{q}^{}\eta_{qp,\;s}I_{p,s};\;\frac{dR_{p,s}}{dt}\;=\;\mu I_{p,s}+\;\sum_{q}^{}\eta_{pq,\;s}R_{q,s}\;-\;\sum_{q}^{}\eta_{qp,\;s}R_{p,s}$$

### (c) Comparing model predictions to past DMV outbreaks

#### Dataset

We used stranding data (records of dolphins washed ashore or found floating) from NOAA (March 2010–September 2014) to capture dolphin mortality before and during the 2013−2014 DMV outbreak and from the Smithsonian Division of Marine Mammal Collections (March 1984–September 1988) for the 1987−1988 DMV outbreak. To isolate DMV-related mortality, we calculated excess mortality by removing the average ‘background’ mortality (such as from natural mortality or human interactions) for each latitude using data from the 3 years prior to each outbreak [22,54]. We also estimated probable infection dates based on the stranding observation date, and the stranding decay status (see electronic supplementary material, §S9) to generate a time series of observed infections, hereafter referred to as ‘outbreak data’.

To compare model infection predictions to outbreak data, we normalized the weekly infection incidence across patches to the maximum incidence value. Model performance was assessed using Pearson’s correlations (higher values indicate more consistent model estimates) and the sum squared errors (SSE) (lower SSE values indicate more consistent model estimates) between modelled and observed infection curves.

#### Determining epidemic onset

Based on NOAA declarations that DMV unusual mortality events began in July 1987 and 2013, with earliest cases between New Jersey and Virginia, we constrained the model start dates between 1 May and 30 June and locations limited to patch 1 or patch 2, to identify the most likely epidemic onset scenario.

#### *Assessing seasonal differences in* β

Using the most likely onset scenario, we then tested two seasonal hypotheses influencing transmission (see electronic supplementary material, §S7.3 for additional methodological details):

(1) Seasonal social behaviour: the PCDP observes a peak in calf births between 15 April and 7 July (electronic supplementary material, figures S4, S5) suggesting heightened breeding activity during this time period. Dolphins tend to form larger groups [55–57] and exhibit more synchronized breathing [58] during breeding activity; since synchrony behaviour can be linked to disease spread [36], we reduced transmission rate ($\beta_{ss^{\prime }}$) outside of 15 April–7 July to reflect a higher synchrony degree ($k_{s}$) during the breeding season.
(2) Seasonal environment: like other enveloped viruses (e.g. SARS-CoV-2, influenza), morbilliviruses probably survive longer in colder conditions and are inactivated by heat [59] making them more efficient at transmitting in colder seasons. Therefore, we reduced transmission rates ($\beta_{ss^{\prime }}$) outside of the cold-water season of January–March to account for lower per contact DMV infectiousness (τ) at warmer temperatures.

A sensitivity analysis tested the impact of altering both the seasonal window lengths and the magnitude of $\beta_{ss`}$ reduction (see electronic supplementary material, §S11.2).

#### Evaluating the role of metapopulation structure on the spread of DMV

Using the best fitting seasonal model determined above, we considered how different components of metapopulation structure impacted the spread of DMV by evaluating the results of three different models in which we controlled variation in: (i) movement, (ii) ecotype movement and (iii) ecotype contact.

(1) Movement control: this control model assumed one $\eta_{pq,E}$ range for all estuarine individuals and one $\eta_{pq,C}$ range for all coastal individuals among all patches by taking the average of the corresponding $\eta_{pq,s}$ values. This model removes the differences in movement rates along the coastline but retains the differences in movement rates between coastal and estuarine ecotypes.
(2) Ecotype movement control: this control model assumes the same $\eta_{pq,s}$ range for both coastal and estuarine individuals between all patches which removes the observed differences in movement rate both along the coastline and by ecotype.
(3) Ecotype contact structure: this control model assumes $\beta_{EE}=\beta_{CC}$ by averaging the two ranges which removes the variability in contact among ecotypes.

### (d) Applying the model to inform preparedness for future DMV epidemics

We use our best fitting model to inform (i) how the epidemic risk of DMV varies depending on the start time and location of a DMV outbreak and (ii) the best patch for establishing surveillance sites for DMV in Tamanend’s bottlenose dolphins.

#### Epidemic risk analysis

Epidemic risk for an infection was calculated beginning in each month of the year (12) in each patch (4) for 48 total scenarios. For each scenario, we simulate 100 epidemics and calculate epidemic risk as the epidemic size (the proportion of individuals that ended up infected across all patches) multiplied by the epidemic probability (the proportion of simulations with an epidemic size was greater than 10%). We also examined the SSE and Pearson’s correlation values for all scenarios to the 2013 outbreak data to determine their similarity to historic DMV outbreaks in Tamanend’s dolphins.

#### Sentinel surveillance analysis

NMFS has established management units of estuarine stocks in patches 2, 3 and 4. Since surveillance methods are more practical to carry out on estuarine stocks than coastal stocks, we examined the difference in weekly infection incidence between the estuarine individuals in these three patches (normalized by the total number of estuarine individuals in the patch) and the infection incidence along the full coastline (normalized by the total number of individuals across patches and ecotypes). The resulting difference is thus a measure of how much estuarine incidence in each patch over or underestimates total coastline incidence; the patch where this difference is closest to zero for all 48 epidemic scenarios would be the best option for establishing a sentinel surveillance site.

## 3. Results

### (a) Metapopulation structure can reproduce historical epidemic dynamics

Using empirical sighting and contact data from the MABDC and the PCDP, we produced novel estimates of Tamanend’s bottlenose dolphin (i) metapopulation patches along the Atlantic coast, (ii) movement rates between these patches and (iii) contact-driven transmission rates within patches. We used these results to model the spread of DMV along the Atlantic coast and compared the resulting infection dynamics to those of past DMV outbreaks to inform mechanisms that drive transmission.

#### (i) Dolphin movement and contact varies by ecotype

Using sighting histories established for 423 dolphins with MABDC data (figure 2A,B), we clustered dolphins based on similar spatiotemporal sightings (figure 2C). We determined the optimal number clusters P is four (electronic supplementary material, figure S3) and thus divided the coastline into four metapopulation patches based on the average warm-water season (defined as July–September; electronic supplementary material, figure S2) habitat ranges of dolphins in each cluster (figure 2D) [25]. The resulting patch 1 occupies the coastlines of the northernmost parts of our study area (New Jersey to northern Virginia), followed by patch 2 (southern Virginia to northern North Carolina), patch 3 (southern North Carolina) and patch 4 (South Carolina and Georgia). A comparison to NOAA’s presumed ranges for Tamanend’s dolphin stocks suggests that our patch delineation is valid (electronic supplementary material, figure S1).

Using MABDC sighting data from dolphins of different ecotypes (coastal *n* = 29; estuarine *n* = 394) across 28 field sites along the Atlantic coast, we estimated movement rates of both coastal $\left(\eta_{pq,C}\right)$ and estuarine $\left(\eta_{pq,E}\right)$ individuals between our defined patches. We found that these movement rates are generally higher for coastal individuals compared to estuarine individuals except between patches 1 and 2. For both estuarine and coastal individuals, the highest rate of movement occurs between patches 3 and 4 (figure 2E; electronic supplementary material, table S5).

Using behavioural data on coastal (*n* = 16) and estuarine (*n* = 55) individuals from the PCDP, we estimated that mean synchrony degree (i.e. the average number of synchronized breathing contacts capable of transmitting DMV) is $k_{C}=$8 individuals per day for coastal individuals and $k_{E}=$6 individuals per day for estuarine individuals. We also empirically estimated synchrony mixing (i.e. the proportion of contacts of one ecotype that are of the other ecotype) between estuarine and coastal individuals (α) to range between 0 and 0.06. We thus estimated the ecotype-specific transmission rates within ($\beta_{CC}$, $\beta_{EE}$) and between ($\beta_{CE}$, $\beta_{EC}$) ecotypes, and predicted transmission rates between coastal individuals $\left(\beta_{CC}=0.256\right)$ to be higher than those between estuarine individuals $\left(\beta_{EE}=0.192\right)$; within ecotype transmission rates were predicted to be higher than between ecotype transmission rates ($\beta_{CE}$ and $\beta_{EC}$ = 0.007; figure 2F).

#### (ii) Seasonal changes in contact best explain past infection dynamics

Based on the metapopulation dynamics and transmission rates we empirically estimated, we predicted epidemic dynamics in Tamanend’s dolphins along the Atlantic coast using an epidemiological metapopulation model (figure 2G). We calculated the SSE and Pearson’s correlations to observed epidemic dynamics and found that model predictions for infected animals over time are most consistent with data from past outbreaks when the outbreak onset is assumed to be in patch 2 between 1 May and 10 May (figure 2A; electronic supplementary material, figure S8).

**Figure 3.**
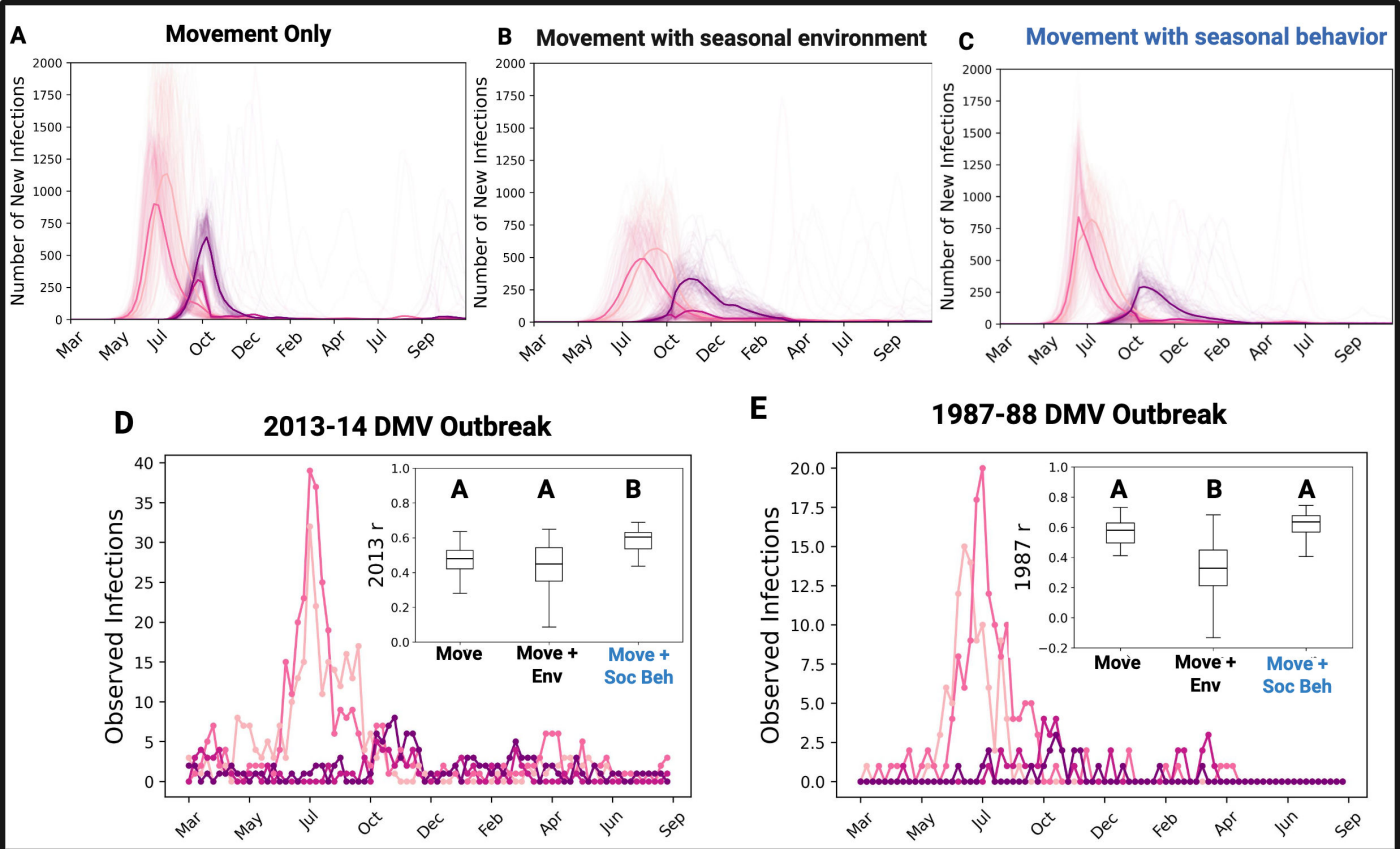
The effect of seasonality in transmission on disease dynamics. The infection time series from an epidemiological model that considers differences in the transmission rate $\left(\beta_{ss^{\prime }}\right)$ among ecotypes and (A) only dolphin movement with no seasonal changes to $\beta_{ss^{\prime }}$; (B) dolphin movement with higher $\beta_{ss^{\prime }}$ in the cold-water season due to the more efficient environmental conditions; and (C) dolphin movement and higher $\beta_{ss^{\prime }}$ in the breeding season due to increased synchrony behaviour. We see that a model with contact structured by seasonal behaviour (C), indicated in blue, is most consistent with DMV outbreak data from the 2013 (D) and 1987 (E) epidemics based on the significantly higher Pearson’s correlation coefficient (*r*) of the model’s infection results to the 2013 stranding data (boxplot insets) and visual comparisons of the time series to both DMV outbreak years. Figure compiled in BioRender (https://BioRender.com/umi60zb).

Next, we tested hypotheses about the impact of seasonal changes due to environmental factors and social behaviour on transmission dynamics. In the first scenario, we considered higher DMV infectiousness in colder environments where enveloped viruses such as DMV are thought to be more effectively transmitted [59]. We find this model is less consistent with the 1987 and 2013 outbreak dynamics (figure 3B,D,E; electronic supplementary material, figure S9). In a second scenario, we considered higher breathing synchrony contact during breeding seasons when contact rates are typically higher [58]. We find this scenario better predicts observed dynamics (figure 3C,E; electronic supplementary material, figure S9) by reducing infection peaks in patches 1, 3 and 4 (figure 3C) as dolphins are most prevalent in these patches when contact is lower, and allowing for a longer epidemic period from the lower transmission outside the short breeding season. Notably, other realistic changes to breeding and cold-water season lengths and transmission rates do not qualitatively affect these results (electronic supplementary material, table S5). This indicates contact structured by seasonal social behaviour may explain the spatial heterogeneity and temporal dynamics of DMV outbreaks.

### (b) Movement and contact structure significantly impacts ecotype vulnerability

Best available data suggest that the coastal ecotype was more vulnerable than the estuarine ecotype [22,25,47] to DMV infection. Our empirically informed epidemiological metapopulation model predicts higher relative infection burden for coastal individuals compared to estuarine individuals, with a larger bias in burden when seasonal social behaviour is incorporated (electronic supplementary material, figure S10).

**Figure 4.**
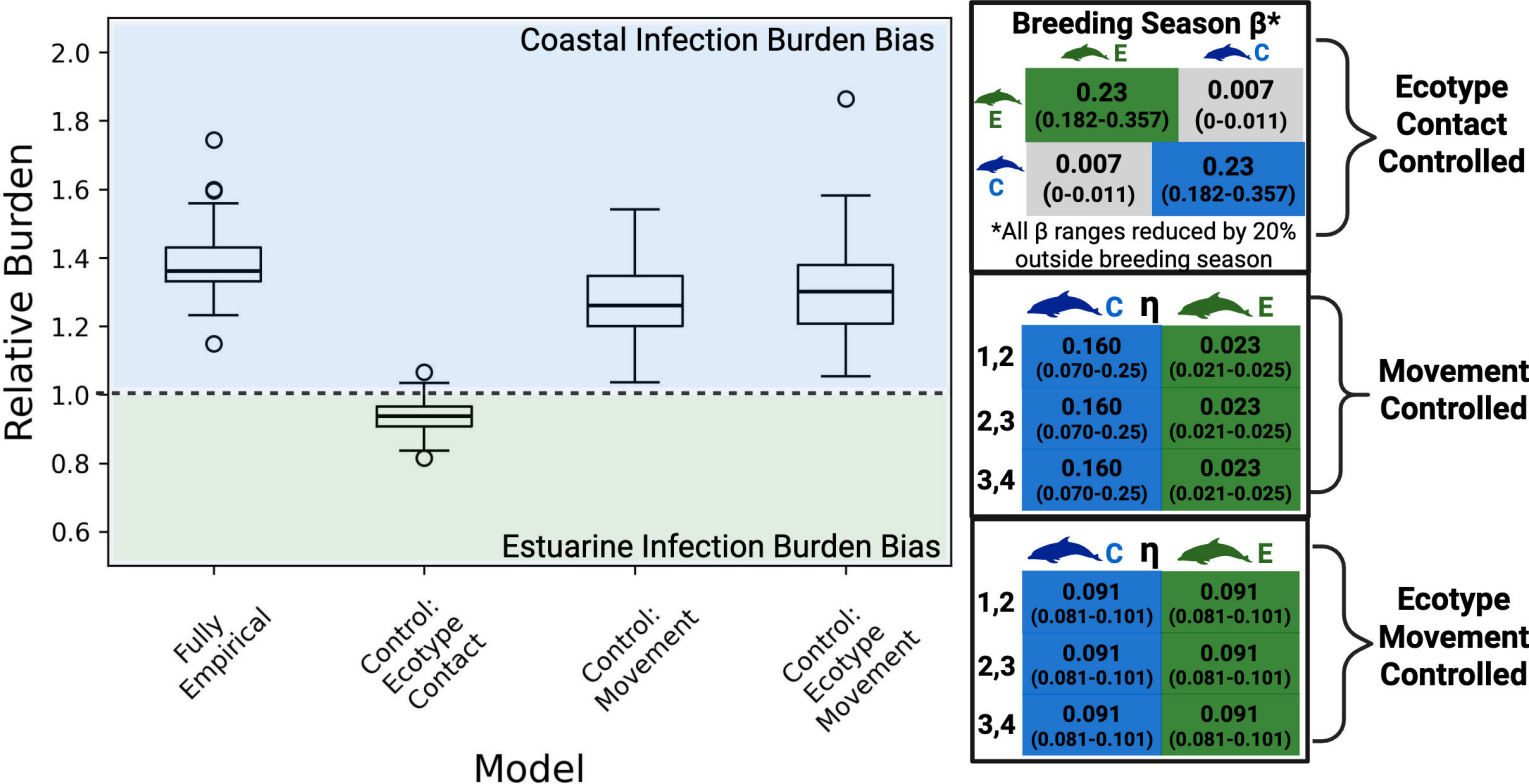
The effect of movement and contact on ecotype infection burden. Relative burden is the ratio of the proportion of coastal individuals infected to the proportion of estuarine individuals infected. When relative burden is equal to 1 (dotted line), there is no bias in infection burden between the two ecotypes. We controlled different components of the metapopulation structure captured in figure 2 (labelled here as fully empirical) to examine their effect on relative burden by removing heterogeneity in $\beta_{ss^{\prime }}$ or $\eta_{pq,s}$ estimates. We found that infection is biased towards coastal individuals in each control scenario except when there is no heterogeneity in $\beta_{ss^{\prime }}$ among ecotypes, suggesting that this heterogeneity may be responsible for the higher coastal infection burdens observed in past DMV outbreaks. Figure compiled in BioRender (https://BioRender.com/o8ajysq).

To mechanistically understand the impact of contact and movement on ecotype vulnerabilities, we systematically controlled and varied different components of the metapopulation structure in our model. When movement rates ($\eta_{pq,s})$ were controlled (i.e. assumed a homogeneous movement rate between patches for each ecotype), the relative infection burden of coastal to estuarine individuals is unaffected (figure 4; electronic supplementary material, figure S11), but infection in patch 1 occurred much later than observed in past epidemics (electronic supplementary material, figures S12 and 13). This was also observed to a lesser degree when movement rates were also controlled by ecotype (i.e. assumed the same homogeneous movement rate across patches and ecotypes) (electronic supplementary material, figures S12 and 13). When ecotype contact structure was controlled (i.e. assumed the same homogeneous transmission rate $\beta_{ss^{\prime }}$ for both ecotypes), the relative disease burden between coastal and estuarine infections was more biased towards estuarine infection (figure 4). We find these results are robust to differences in the number of estuarine individuals in each patch (electronic supplementary material, §S11.1, figure S7). Our findings thus suggest that spatial and ecotype heterogeneity in movement are important for describing infection dynamics at epidemic onset, while ecotype heterogeneity in contact may explain higher coastal ecotype vulnerability to DMV along the coastline.

### (c) Disease surveillance and epidemic risk assessment is optimized by leveraging movement and contact structure

To inform outbreak preparedness and optimize surveillance for future DMV outbreaks, we examined how epidemic risk (a combination of the size of the infected population and the likelihood of an epidemic occurring) varies with the onset location and time, and how particular populations along the coast might serve as sentinels for DMV surveillance.

**Figure 5.**
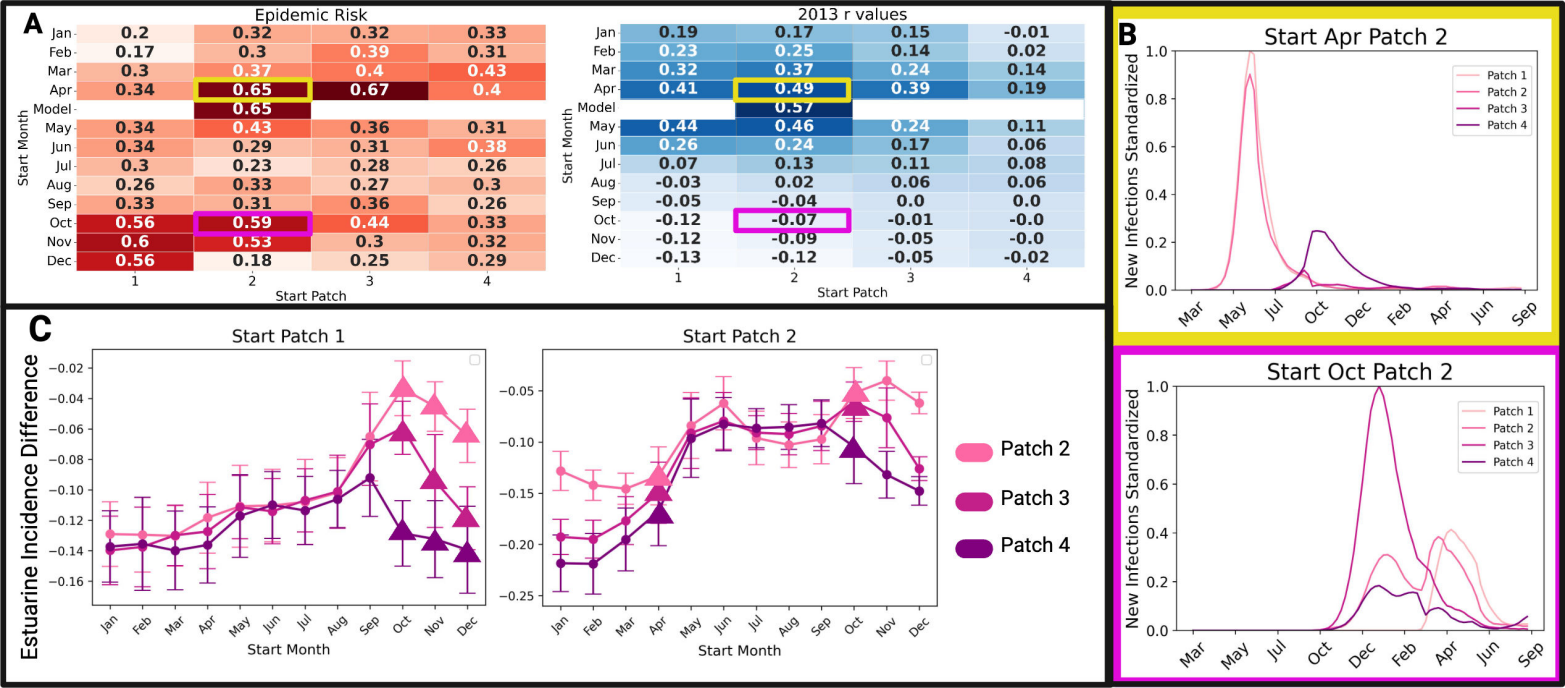
Applying the model to inform DMV preparedness. (A) The epidemic risk values for DMV infection beginning in all possible onset scenarios and their associated Pearson’s correlation coefficients (r) to the 2013 outbreak data. These values show that outbreaks beginning in patches 1 and 2 have the highest epidemic risk, but not all of these scenarios result in the dynamics observed in past outbreaks as indicated by their low *r* values. For example, (B) compares two high-risk epidemic scenarios, showing that outbreaks starting in migratory season 1 (yellow) more closely resemble historic DMV epidemics than those beginning in migratory season 2 (pink). (C) To identify the optimal patch for DMV sentinel surveillance, we compared weekly infection rates among estuarine individuals in patches 2, 3 and 4 against the entire coastline’s rates for outbreaks starting in high-risk patches (1, left and 2, right). Values near zero indicate the patch best represents coastline incidence, with patch 2 emerging as optimal. Triangles mark high epidemic risk scenarios from (A), with error bars showing standard deviation. Figure compiled in BioRender (https://BioRender.com/tax8dem).

The epidemic risk of our seasonal social behaviour model where infection begins between 1 May−10 May in patch 2 was 65%. Out of 48 alternative onset location and time scenarios, only six resulted in similar (55–75%) epidemic risk. Of these similar scenarios, most supported epidemic onset during the migratory seasons in patches 1 or 2 and no scenario with epidemic onset in patch 4 or during the warm (July–September) or cold (January–March) water seasons carried the same epidemic risk (figure 5A). Furthermore, high epidemic risk scenarios with onset in migratory season 1 (April–June) resulted in epidemic dynamics similar to past DMV outbreaks compared to high-risk scenarios with onset in migratory season 2 (October–December) (figure 5A,B; electronic supplementary material, figure S14).

While it is assumed that coastal populations bore a higher infection burden in past DMV outbreaks, estuarine individuals are more accessible for data collection given their smaller, predictable and nearshore habitat ranges. Thus, we examined the possibility of estuarine populations serving as sentinels for DMV monitoring for the entire Atlantic coast. We compared the average weekly infection incidence of estuarine individuals (in patches 2, 3 and 4), as these patches currently have established estuarine populations [25], to the average weekly infection incidence for the entire coastline (coastal + estuarine incidence in all four patches) for the 48 onset scenarios. Our results demonstrate that estuarine incidence in each patch tends to underestimate the entire coastline incidence, but estuarine populations in patch 2 are most representative (figure 5C; electronic supplementary material, figure S15). This is particularly the case for the seven high-risk scenarios. Thus, we suggest that sentinel surveillance for DMV would be most effective if carried out in estuarine individuals occupying patch 2.

## 4. Discussion

Our data-driven estimates of both movement and contact rates in Tamanend’s bottlenose dolphins highlight differences in behaviour along the coastline and inform the role of metapopulation structure on their vulnerability to infectious disease threats. We find that individual movement along the Atlantic coast differs spatially and by ecotype. We also highlight the use of our model in optimizing epidemic surveillance and risk assessments in this vulnerable population.

We find higher coastal movement rates between southern metapopulation patches than the northern patches, likely reflecting seasonal stock presence. For example, while only one migratory coastal stock is thought to move between patches 1 and 2, two are thought to move between the southern patches [25]. Coastal movement rates also show greater variation than estuarine, possibly due to a smaller coastal sample size, or temporal ecotype differences, as estuarine populations tend to engage in a more year-round dispersal while coastal populations engage in seasonal migration [25]. Notably, we found that slower coastal movement in the north is important in reproducing observed DMV infection; if coastal individuals reach the northern coast (e.g. Delaware and New Jersey) too quickly, infection that originated further south (e.g. Virginia or North Carolina) is not able to reach this area. This suggests that migration rates strongly influence epidemic outcomes. Consequently, recent poleward shifts in odontocete distributions in the northeastern United States [60,61] could indicate increased northern migration, potentially altering the dynamics of contagious infections.

We find that increased breathing synchrony during the breeding season produced epidemic dynamics consistent with past outbreaks. This contact behaviour, critical for respiratory disease spread in cetaceans and influenced by age and sex class [36], is higher in coastal than estuarine individuals helping explain a higher coastal infection burden. Several factors may contribute to this difference. First, coastal population [25] and group sizes [53] are larger than estuarine estimates; coastal population sizes number in the thousands, while estuarine estimates are in the hundreds [25], potentially increasing contact rates under density-dependent transmission. Second, coastal populations have larger habitat ranges [25] and thus spend more time travelling in groups with greater opportunity for synchronized breathing. Furthermore, offshore foraging on large schooling fish may promote more social foraging behaviours not as common in estuarine environments where it is often more advantageous to be solitary [62]. While NOAA has declared two coastal stocks depleted due to DMV [39], no estuarine stocks hold this status. However, our model predicts nearly 50% infection across estuarine populations. The true effect of DMV on estuarine dolphins is unclear [25,47], but critical to assess, as many mid-Atlantic estuarine stocks are considered strategic due to the impact of fisheries interactions [25]. Enhanced surveillance for DMV antibodies in estuarine dolphins [26] could clarify these impacts.

Our model highlights the influence of metapopulation structure on disease dynamics and offers a tool for improving preparedness for future outbreaks. When simulating outbreaks with different starting times and locations, we found that only seven scenarios matched the epidemic risk of historic DMV events, most originating at northern latitudes. This suggests that dolphin behaviour may structure northern emergence hotspots, helping explain the similar dynamics of the 1987 and 2013 outbreaks. Individuals at northern latitudes may also face higher risk of initial infection due to more interactions with other morbillivirus reservoir species, such as long-finned pilot whales (*Globicephala melas*) [63,64]. Notably, only scenarios that began in migratory season 1 (April–June) replicated historical DMV dynamics. While four migratory season 2 (October–December) scenarios also showed high epidemic risk, they produced different dynamics with epidemic peaks in the south during the cold-water season. This has important implications for targeted control and surveillance strategies, highlighting when and where measures might be most effective.

While the control of respiratory disease spread in dolphins remains a challenge, surveillance of DMV in Tamanend’s dolphins is feasible through boat-based biopsies [26] particularly in individuals of estuarine ecotype given their spatial ecology. Our work shows that estuarine individuals in patch 2 (i.e. individuals in the northern North Carolina estuarine stock) would be optimal sentinels for routine DMV surveillance for all Tamanend’s dolphins during or following an outbreak. Indeed, past DMV surveillance work carried out in Georgia (our patch 4) showed a low proportion of estuarine individuals infected compared to coastal individuals [26], supporting our finding that estuarine stocks in this region may not be the best indicators of infection during or post-epidemic.

Metapopulation disease models parameterized with fine-scale host movement patterns have been theoretically illustrated to be critical in predicting geographically heterogeneous disease dynamics [65]. As our model captures spatial and ecotype heterogeneity in movement and contact among a complex marine species, we demonstrate that such empirically informed structured models indeed reproduce observed dynamics compared to models without such variation. This suggests that such biological processes are critically important to consider for understanding disease spread. Our results also provide empirical support for theoretical metapopulation disease models, which show that while highly contagious diseases can spread widely through well connected metapopulations [66], infection tends to decline in subsequent patches relative to the origin patch [67]. Indeed, while infection spreads to all patches in our model, the highest number of infections occur in the origin patch (2) with declining infection rates into the subsequent patches.

Our model can be expanded to forecast future epidemics under a range of scenarios. First, using a collated dataset such as the MABDC allows for ongoing updates as researchers contribute new sighting data. For example, our coastal sample size is small relative to population estimates (electronic supplementary material, table S3); incorporating new data from current research sites could refine movement estimates or support finer spatial resolutions [50]. Additionally, while the Florida coastline was affected by DMV [22], limited movement data in this region prevent us from fully capturing infection dynamics there. While our supplemental analysis suggests migration into Florida, empirical data would enable direct model inclusion. Second, we can forecast outbreak timing by incorporating dolphin life history traits such as natural birth and mortality rates [68,69]. Epidemics end when too few susceptible individuals remain, but births replenish this pool, enabling recurrent outbreaks [70]. The 26-year gap between the DMV outbreaks in Tamanend’s dolphins [21,22] and similar lags in other dolphin [18] and seal [71] populations highlights the importance of predicting these interepidemic periods. Third, our model can incorporate behavioural changes during infection such as avoidance [72], or sickness behaviours [73]. For example, during the 2013 outbreak, network connectedness was reduced in Tamanend’s dolphins in Florida, which probably mitigated infection in this region [27]. Fourth, we can account for spatial variation in ecotype mixing. Although our model assumes uniform mixing, data shows there is likely variation. For instance, in Florida, suspected estuarine and coastal dolphins overlap more during warm and cold-water seasons compared to migratory seasons [27], with higher overlap than observed in Georgia [26]. Finally, this model can integrate the impacts of global change. Climate-driven stressors such as warming oceans can increase disease susceptibility [74] and alter seasonal connectivity and distribution [60]. Adjusting parameters to reflect such changes enables predictions of disease consequences in Tamanend’s dolphins and related species [75,76]. As the biodiversity crisis grows [77], such models are increasingly vital, particularly for wide-ranging marine mammals capable of cross-species virus transmission [78,79], and are critical tools in the arsenal of sustained efforts to mitigate these impacts.

## Data Availability

All data and code needed to reproduce this analysis are available at the following repository [80]. Supplementary material is available online [81].

## References

[1] McCallum H, Dobson A. 1995 Detecting disease and parasite threats to endangered species and ecosystems. Trends Ecol. Evol. **10**, 190–194. (10.1016/s0169-5347(00)89050-3) 21237000

[2] Daszak P, Cunningham AA, Hyatt AD. 2000 Emerging infectious diseases of wildlife: threats to biodiversity and human health. Science **287**, 443–449. (10.1126/science.287.5452.443) 10642539

[3] Bossard GD. 2006 Marine mammals as sentinel species for ocean and human health. Oceanography **19**, 134–137. (10.5670/oceanog.2006.77) 21160025

[4] Heithaus MR, Frid A, Wirsing AJ, Worm B. 2008 Predicting ecological consequences of marine top predator declines. Trends Ecol. Evol. **23**, 202–210. (10.1016/j.tree.2008.01.003) 18308421

[5] Moore SE. 2008 Marine mammals as ecosystem sentinels. J. Mammal. **89**, 534–540. (10.1644/07-MAMM-S-312R1.1)

[6] Sanderson CE, Alexander KA. 2020 Unchartered waters: climate change likely to intensify infectious disease outbreaks causing mass mortality events in marine mammals. Glob. Chang. Biol. **26**, 4284–4301. (10.1111/gcb.15163) 32558115

[7] Altizer S, Bartel R, Han BA. 2011 Animal migration and infectious disease risk. Science **331**, 296–303. (10.1126/science.1194694) 21252339

[8] Sah P, Mann J, Bansal S. 2018 Disease implications of animal social network structure: a synthesis across social systems. J. Anim. Ecol. **87**, 1–13. (10.1111/1365-2656.12786) 29247466

[9] Cassirer EF *et al*. 2018 Pneumonia in bighorn sheep: risk and resilience. J. Wildl. Manag. **82**, 32–45. (10.1002/jwmg.21309)

[10] Blanchong JA, Anderson CA, Clark NJ, Klaver RW, Plummer PJ, Cox M, McAdoo C, Wolff PL. 2018 Respiratory disease, behavior, and survival of mountain goat kids. J. Wildl. Manag. **82**, 1243–1251. (10.1002/jwmg.21470)

[11] Hochachka WM, Dhondt AA. 2000 Density-dependent decline of host abundance resulting from a new infectious disease. Proc. Natl Acad. Sci. USA **97**, 5303–5306. (10.1073/pnas.080551197) 10792031 PMC25823

[12] O’Dea MA, Jackson B, Jackson C, Xavier P, Warren K. 2016 Discovery and partial genomic characterisation of a novel nidovirus associated with respiratory disease in wild shingleback lizards (Tiliqua rugosa). PLoS One **11**, e0165209. (10.1371/journal.pone.0165209) 27828982 PMC5102451

[13] Sumithra TG, Chaturvedi VK, Susan C, Siju SJ, Rai AK, Harish C, Sunita SC. 2013 Mycoplasmosis in wildlife: a review. Eur. J. Wildl. Res. **59**, 769–781. (10.1007/s10344-013-0769-9)

[14] Leung NHL. 2021 Transmissibility and transmission of respiratory viruses. Nat. Rev. Microbiol. **19**, 528–545. (10.1038/s41579-021-00535-6) 33753932 PMC7982882

[15] Duignan PJ *et al*. 2014 Phocine distemper virus: current knowledge and future directions. Viruses **6**, 5093–5134. (10.3390/v6125093) 25533658 PMC4276944

[16] Van Bressem MF *et al*. 2014 Cetacean morbillivirus: current knowledge and future directions. Viruses **6**, 5145–5181. (10.3390/v6125145) 25533660 PMC4276946

[17] Aguilar A, Raga JA. 1993 The striped dolphin epizootic in the Mediterranean Sea. Ambio **22**, 524–528.

[18] Keck N *et al*. 2010 Resurgence of Morbillivirus infection in Mediterranean dolphins off the French coast. Vet. Rec. **166**, 654–655. (10.1136/vr.b4837) 20495168

[19] Rubio-Guerri C, Melero M, Esperón F, Bellière E, Arbelo M, Crespo J, Sierra E, García-Párraga D, Sánchez-Vizcaíno J. 2013 Unusual striped dolphin mass mortality episode related to cetacean morbillivirus in the Spanish Mediterranean Sea. BMC Vet. Res. **9**, 106. (10.1186/1746-6148-9-106) 23702190 PMC3666923

[20] Costa APB, Mcfee W, Wilcox LA, Archer FI, Rosel PE. 2022 The common bottlenose dolphin (Tursiops truncatus) ecotypes of the western North Atlantic revisited: an integrative taxonomic investigation supports the presence of distinct species. Zool. J. Linn. Soc. **196**, 1608–1636. (10.1093/zoolinnean/zlac025)

[21] Lipscomb TP, Schulman FY, Moffett D, Kennedy S. 1994 Morbilliviral disease in Atlantic bottlenose dolphins (Tursiops truncatus) from the 1987-1988 epizootic. J. Wildl. Dis. **30**, 567–571. (10.7589/0090-3558-30.4.567) 7760492

[22] Morris SE, Zelner JL, Fauquier DA, Rowles TK, Rosel PE, Gulland F, Grenfell BT. 2015 Partially observed epidemics in wildlife hosts: modelling an outbreak of dolphin morbillivirus in the northwestern Atlantic, June 2013–2014. J. R. Soc. Interface **12**, 20150676. (10.1098/rsif.2015.0676) 26577594 PMC4685842

[23] Norman SA *et al*. 2012 The application of GIS and spatiotemporal analyses to investigations of unusual marine mammal strandings and mortality events. Mar. Mammal Sci. **28**, E251–E266. (10.1111/j.1748-7692.2011.00507.x)

[24] Dougherty ER, Seidel DP, Carlson CJ, Spiegel O, Getz WM. 2018 Going through the motions: incorporating movement analyses into disease research. Ecol. Lett. **21**, 588–604. (10.1111/ele.12917) 29446237

[25] Hayes SA. 2023 US Atlantic and Gulf of Mexico marine mammal stock assessments 2022. NOAA Technical Memorandum NMFS-NE-304. US Department of Commerce, NOAA, NMFS, NEFSC.

[26] Balmer B *et al*. 2018 Ranging patterns, spatial overlap, and association with dolphin morbillivirus exposure in common bottlenose dolphins (Tursiops truncatus) along the Georgia, USA coast. Ecol. Evol. **8**, 12890–12904. (10.1002/ece3.4727) 30619591 PMC6308875

[27] Szott EA, Brightwell K, Gibson Q. 2022 Assessment of social mixing and spatial overlap as a pathway for disease transmission in a northeast Florida estuarine dolphin community. Mamm. Biol. **102**, 1267–1283. (10.1007/s42991-022-00282-y)

[28] Weber N, Carter SP, Dall SRX, Delahay RJ, McDonald JL, Bearhop S, McDonald RA. 2013 Badger social networks correlate with tuberculosis infection. Curr. Biol. **23**, R915–R916. (10.1016/j.cub.2013.09.011) 24156807

[29] Hamede RK, Bashford J, McCallum H, Jones M. 2009 Contact networks in a wild Tasmanian devil (Sarcophilus harrisii) population: using social network analysis to reveal seasonal variability in social behaviour and its implications for transmission of devil facial tumour disease. Ecol. Lett. **12**, 1147–1157. (10.1111/j.1461-0248.2009.01370.x) 19694783

[30] Rendell L, Cantor M, Gero S, Whitehead H, Mann J. 2019 Causes and consequences of female centrality in cetacean societies. Phil. Trans. R. Soc. B **374**, 20180066. (10.1098/rstb.2018.0066) 31303160 PMC6664132

[31] Wells RS, Scott MD, Irvine AB. 1987 The social structure of free-ranging bottlenose dolphins. In Current mammalogy, vol. 1 (ed. H Genoways), pp. 247–305. London, UK: Plenum Press. (10.1007/978-1-4757-9909-5_7)

[32] Mann J. 1999 Behavioral sampling methods for cetaceans: a review and critique. Mar. Mammal Sci. **15**, 102–122.

[33] Bräger S. 1993 Diurnal and seasonal behavior patterns of bottlenose dolphins (Tursiops truncatus). Mar. Mammal Sci. **9**, 434–438. (10.1111/j.1748-7692.1993.tb00477.x)

[34] Sakai M, Morisaka T, Kogi K, Hishii T, Kohshima S. 2009 Fine-scale analysis of synchronous breathing in wild Indo-Pacific bottlenose dolphins (Tursiops aduncus). Behav. Process **83**, 48–53. (10.1016/j.beproc.2009.10.001) 19850113

[35] Moller LM, Harcourt RG. 1998 Social dynamics and activity patterns of bottlenose dolphins, Tursiops truncatus. Proc. Linneaen Soc. New South Wales **120**, 181–189.

[36] Collier MA *et al*. 2025 Breathing synchrony shapes respiratory disease risk in bottlenose dolphins. Commun. Biol. **8**, 870. (10.1038/s42003-025-08161-1) 40473771 PMC12141649

[37] NOAA Fisheries 2024 2013-2015 Bottlenose dolphin unusual mortality event in the mid-Atlantic (closed). Marine Life in Distress. See https://www.fisheries.noaa.gov/national/marine-life-distress/2013-2015-bottlenose-dolphin-unusual-mortality-event-mid-atlantic (accessed 8 July 2025).

[38] McLellan WA, Friedlaender AS, Mead JG, Potter CW, Pabst DA. 2002 Analysing 25 years of bottlenose dolphin (Tursiops truncatus) strandings along the Atlantic coast of the USA: do historic records support the coastal migratory stock hypothesis? J. Cetacean Res **4**, 297–304. (10.47536/jcrm.v4i3.843)

[39] NOAA Fisheries. 1993 Depleted designation for western North Atlantic coastal migratory stock of bottlenose dolphins. See https://www.fisheries.noaa.gov/action/depleted-designation-western-north-atlantic-coastal-migratory-stock-bottlenose-dolphins.

[40] Waring GT, Josephson E, Maze-Foley K, Rosel PE. 2016 US Atlantic and Gulf of Mexico marine mammal stock assessments 2015. NOAA Tech Memo NMFS NE 238. US Department of Commerce, NOAA, NMFS, NEFSC.

[41] White LA, Forester JD, Craft ME. 2018 Dynamic, spatial models of parasite transmission in wildlife: their structure, applications and remaining challenges. J. Anim. Ecol. **87**, 559–580. (10.1111/1365-2656.12761) 28944450

[42] Colombi D, Serra-Cobo J, Métras R, Apolloni A, Poletto C, López-Roig M, Bourhy H, Colizza V. 2019 Mechanisms for lyssavirus persistence in non-synanthropic bats in Europe: insights from a modeling study. Sci. Rep. **9**, 537. (10.1038/s41598-018-36485-y) 30679459 PMC6345892

[43] Langwig KE *et al*. 2021 Mobility and infectiousness in the spatial spread of an emerging fungal pathogen. J. Anim. Ecol. **90**, 1134–1141. (10.1111/1365-2656.13439) 33550607 PMC8248334

[44] Fulford GR, Roberts MG, Heesterbeek JAP. 2002 The metapopulation dynamics of an infectious disease: tuberculosis in possums. Theor. Popul. Biol. **61**, 15–29. (10.1006/tpbi.2001.1553) 11895380

[45] Brandell EE, Dobson AP, Hudson PJ, Cross PC, Smith DW. 2021 A metapopulation model of social group dynamics and disease applied to Yellowstone wolves. Proc. Natl Acad. Sci. USA **118**, e2020023118. (10.1073/pnas.2020023118) 33649227 PMC7958402

[46] Swinton J, Harwood J, Grenfell BT, Gilligan CA. 1998 Persistence thresholds for phocine distemper virus infection in harbour seal Phoca vitulina metapopulations. J. Anim. Ecol. **67**, 54–68. (10.1046/j.1365-2656.1998.00176.x)

[47] Scott GP, Burn DM, Hansen LJ. 1988 The dolphin dieoff: long-term effects and recovery of the population. In OCEANS ’88: a partnership of marine interests, pp. 819–823. Baltimore, MD: IEEE. (10.1109/OCEANS.1988.794905)

[48] Urian K, Hohn AA, Hansen LJ. 1999 Status of the photo-identification catalog of coastal bottlenose dolphins of the western north atlantic: report of a workshop of catalog contributors. NOAA Technical Memorandum NMFS SEFSC 425. US Department of Commerce, NOAA, NMFS, SEFSC.

[49] Hammond P, Mizroch S, Donovan G. 1990 Individual recognition of cetaceans: use of photo-identification and other techniques to estimate population parameters. International Whaling Commission.

[50] Rushing CS. 2023 An ecologist’s introduction to continuous‐time multi‐state models for capture–recapture data. J. Anim. Ecol. **92**, 936–944. (10.1111/1365-2656.13902) 36785976

[51] Karniski C, Patterson EM, Krzyszczyk E, Foroughirad V, Stanton MA, Mann J. 2015 A comparison of survey and focal follow methods for estimating individual activity budgets of cetaceans. Mar. Mammal Sci. **31**, 839–852. (10.1111/mms.12198)

[52] Toth JL, Hohn AA, Able KW, Gorgone AM. 2012 Defining bottlenose dolphin (Tursiops truncatus) stocks based on environmental, physical, and behavioral characteristics. Mar. Mammal Sci. **28**, 461–478. (10.1111/j.1748-7692.2011.00497.x)

[53] Read AJ *et al*. 2013 Stock discrimination of bottlenose dolphins along the outer banks of North Carolina: implications for the risk of entanglement in coastal gill net fisheries. 10 DMM 01. North Carolina Sea Grant Bycatch Reduction Marine Mammal Project.

[54] Karlinsky A, Kobak D. 2021 Tracking excess mortality across countries during the COVID-19 pandemic with the World Mortality Dataset. eLife **10**, e69336. (10.7554/elife.69336) 34190045 PMC8331176

[55] Mann J, Connor RC, Barre LM, Heithaus M. 2000 Female reproductive success in bottlenose dolphins (Tursiops sp.): life history, habitat, provisioning, and group-size effects. Behav. Ecol. **11**, 210–219. (10.1093/beheco/11.2.210)

[56] Daura-Jorge FG, Wedekin LL, Piacentini V de Q, Simões-Lopes PC. 2005 Seasonal and daily patterns of group size, cohesion and activity of the estuarine dolphin, Sotalia guianensis (P.J. van Bénéden) (Cetacea, Delphinidae), in southern Brazil. Rev. Bras. Zool. **22**, 1014–1021. (10.1590/S0101-81752005000400029) 17505744

[57] Karczmarski L, Cockcroft VG, McLachlan A. 1999 Group size and seasonal pattern of occurrence of humpback dolphins Sousa chinensis in Algoa Bay, South Africa. South Afr. J. Mar. Sci. **21**, 89–97. (10.2989/025776199784126024)

[58] Connor RC, Smolker R, Bejder L. 2006 Synchrony, social behaviour and alliance affiliation in Indian Ocean bottlenose dolphins, Tursiops aduncus. Anim. Behav. **72**, 1371–1378. (10.1016/j.anbehav.2006.03.014)

[59] Rijks JM, Osterhaus A, Kuiken T, Frölich K. 2012 Morbillivirus infections. In Infectious diseases of wild mammals and birds in europe (eds D Gaivier-Widen, A Meredith, JP Duff), pp. 99–118. Hoboken, NJ: Wiley-Blackwell. (10.1002/9781118342442)

[60] van Weelden C, Towers JR, Bosker T. 2021 Impacts of climate change on cetacean distribution, habitat and migration. Clim. Chang. Ecol. **1**, 100009. (10.1016/j.ecochg.2021.100009)

[61] Thorne LH, Heywood EI, Hirtle NO. 2022 Rapid restructuring of the odontocete community in an ocean warming hotspot. Glob. Chang. Biol. **28**, 6524–6540. (10.1111/gcb.16382) 36054792 PMC9804436

[62] Roberts SM *et al*. 2023 Tight spatial coupling of a marine predator with soniferous fishes: using joint modelling to aid in ecosystem approaches to management. Divers. Distrib. **29**, 1074–1089. (10.1111/ddi.13746)

[63] Bressem MF, Jepson P, Barrett T. 1998 Further insight on the epidemiology of cetacean morbillivirus in the Northeastern Atlantic. Mar. Mammal Sci. **14**, 605–613.

[64] Duignan PJ *et al*. 1995 Morbillivirus infection in two species of pilot whale (Globicephala sp.) from the western Atlantic. Mar. Mammal Sci. **11**, 150–162. (10.1111/j.1748-7692.1995.tb00514.x)

[65] Daversa DR, Fenton A, Dell AI, Garner TWJ, Manica A. 2017 Infections on the move: how transient phases of host movement influence disease spread. Proc. R. Soc. B **284**, 20171807. (10.1098/rspb.2017.1807) PMC5745403 29263283

[66] Hess G. 1996 Disease in metapopulation models: implications for conservation. Ecology **77**, 1617–1632.

[67] Michalska-Smith M, VanderWaal K, Craft ME. 2022 Asymmetric host movement reshapes local disease dynamics in metapopulations. Sci. Rep. **12**, 9365. (10.1038/s41598-022-12774-5) 35672422 PMC9171740

[68] Ben-Horin T. 2020 Modelling marine diseases. In Marine disease ecology (eds DC Behringer, BR Silliman, KD Lafferty), pp. 233–256. Oxford, UK: Oxford University Press. (10.1093/oso/9780198821632.003.0012)

[69] Keeling MJ, Rohani P. 2008 Modeling infectious disease. Princeton, NJ: Princeton University Press.

[70] Grenfell B, Keeling M. 2007 Dynamics of infectious disease. In Theoretical ecology: principles and applications (eds R May, A McLean), pp. 132–147. Oxford, UK: Oxford University Press. (10.1093/oso/9780199209989.003.0013)

[71] Härkönen T *et al*. 2006 The 1988 and 2002 phocine distemper virus epidemics in European harbour seals. Dis. Aquat. Org. **68**, 115–130. (10.3354/dao068115) 16532603

[72] Stockmaier S, Ulrich Y, Albery GF, Cremer S, Lopes PC. 2023 Behavioural defences against parasites across host social structures. Funct. Ecol. **37**, 809–820. (10.1111/1365-2435.14310)

[73] Lopes PC, Block P, König B. 2016 Infection-induced behavioural changes reduce connectivity and the potential for disease spread in wild mice contact networks. Sci. Rep. **6**, 1–10. (10.1038/srep31790) 27548906 PMC4993150

[74] Learmonth JA, MacLeod C, Santos M, Pierce G, Crick H, Robinson R. 2006 Potential effects of climate change on marine mammals. In Oceanography and marine biology: an annual review, vol. 44 (eds JDM Gordon, RN Gibson, JA Atkinson), pp. 431–464. Boca Raton, FL: CRC Press. (10.1201/9781420006391.ch8)

[75] Dickinson ER, McFarland C, Toïgo C, Michael Scantlebury D, Stephens PA, Marks NJ, Morgan ER. 2024 Host movement dominates the predicted effects of climate change on parasite transmission between wild and domestic mountain ungulates. R. Soc. Open Sci. **11**, 230469. (10.1098/rsos.230469) 38179074 PMC10762430

[76] Gulland FMD, Baker JD, Howe M, LaBrecque E, Leach L, Moore SE, Reeves RR, Thomas PO. 2022 A review of climate change effects on marine mammals in United States waters: past predictions, observed impacts, current research and conservation imperatives. Clim. Chang. Ecol. **3**, 100054. (10.1016/j.ecochg.2022.100054)

[77] Kim K, Dobson AP, Gulland FMD, Harvell CD. 2005 Diseases and the conservation of marine biodiversity. In Marine conservation biology (eds EA Norse, LB Crowder), pp. 149–163. Washington, DC: Island Press.

[78] Gulland FMD, Hall AJ. 2007 Is marine mammal health deteriorating? Trends in the global reporting of marine mammal disease. EcoHealth **4**, 135–150. (10.1007/s10393-007-0097-1)

[79] Simeone CA, Gulland FMD, Norris T, Rowles TK. 2015 A systematic review of changes in marine mammal health in North America, 1972-2012: the need for a novel integrated approach. PLoS One **10**, e0142105. (10.1371/journal.pone.0142105) 26579715 PMC4651562

[80] Collier M. 2025 bansallab/dolphin_metapop: dolphin metapopulation code (Version v1). Zenodo. (10.5281/zenodo.15784437)

[81] Collier MA, Urian K, Theisen S, Jacoby AM, Wilkin S, Patterson EM *et al*. 2025 Supplementary material from: Seasonal contact and migration structure mass epidemics and inform outbreak preparedness in a vulnerable marine mammal. Figshare (10.6084/m9.figshare.c.7943397) PMC12308530 40735844

